# NIH funding for the pediatric surgeon-scientist: A stable entity, but for how long?

**DOI:** 10.1016/j.sopen.2025.02.008

**Published:** 2025-03-04

**Authors:** Troy A. Markel

**Affiliations:** Department of Surgery, Section of Pediatric Surgery, Indiana University School of Medicine, Indianapolis, IN, United States of America; Riley Hospital for Children at Indiana University Health, Indianapolis, IN, United States of America

## Introduction

In the evolving landscape of medical research funding, pediatric surgeons have shown remarkable resilience, successfully securing increasing funds from the National Institutes of Health (NIH) despite broader reports of decreasing funding for surgeons. This trend highlights the critical importance of pediatric surgery research and the dedication of its practitioners to advancing the field.

## The current funding climate

Across the United States, surgeons have faced significant challenges in obtaining research funding from the NIH. Budget constraints and shifting priorities have led to a decrease in available funds for surgical research in general [1]. Many adult surgical specialties have reported difficulties in acquiring the necessary grants to support their innovative projects and clinical trials. A 2018 study looking at NIH grants over a 10-year period (2006–2016) noted that while the number of NIH grant applications increased by nearly 20 %, the success of acquiring these grants decreased by nearly the same amount. The study determined that the number of surgeons as principal investigators was significantly lower than the mean NIH funding rate [1].

Despite reports of decreased funding to surgeons, accurately assessing the current funding environment is actually quite challenging. Recent studies of NIH funding to surgeon scientists have drawn disparate conclusions, mainly due to differences in methodology and inclusion criteria. For example, one study reported a marginal increase in NIH funding to surgeon scientists when comparing years 2010 to 2020 [2], while other studies have suggested no change [3], or even a decrease in NIH grants to surgery departments between 2003 and 2013 [3]. The number of grants awarded to surgeon-scientists also appears to differ across surgical subspecialties, suggesting that funding trends or characteristics of surgeon-scientists vary across different specialties [1].

A recent study by Nguyen et al. surveyed NIH funding to departments of surgery over a 25-year period. They noted that the number of NIH funded investigators in surgical departments increased 1.9 fold from 968 to 1874 investigators. This corresponded to a 4.0 fold increase in total funding of $214 million in 1995 to $861 million in 2020. Despite these encouraging results, the study also showed that the funding gap between surgeon scientists and PhD scientists increased substantially, from $73 million in 1995 to $208 million in 2020 in favor of the PhD scientists [9]. This would either suggest that PhD scientists are more successful than surgeon scientists at obtaining funding, or that departments of surgery are hiring more PhD scientists to offset the declining funding return from its surgeon scientists.

## Challenges for surgeons

Surgeons, who often juggle demanding clinical schedules with research responsibilities, are particularly affected by federal funding challenges. The rigorous process of grant application and the competitive nature of NIH funding further exacerbate the difficulties. Surgeons often face significant challenges in pursuing a successful research career due to heavy clinical demands, limited time for research activities, difficulties securing funding, a lack of dedicated research training, and pressure to maintain high clinical productivity, making it difficult to balance both aspects effectively [4]. In addition, declining earning potential and decreased reimbursement for patient care in the face of increased costs of care and the ever-expanding use of novel expensive technologies and treatments mandate that surgeons spend more time in the operating room to generate revenue. For many surgeons, these challenges have meant scaling back on research endeavors or seeking alternative sources of funding. Pressure to be clinically productive, excessive administrative responsibilities, difficulty obtaining extramural funding, and desire for work-life balance have all been identified as barriers to successful research careers [4].

## Pediatric surgeons: a stable research community

Contrary to the general trend, pediatric surgeons actually reported the highest rates of NIH funding of all surgical subspecialties and the lowest rates of perceived external pressures related to clinical demands, administrative duties, and work-life balance concerns [5]. This correlated to pediatric surgeons having the third highest amount of funding of all general surgery trained subspecialties in 2020, ranking only behind surgical oncology and transplant surgery [2].

In 2024, Shah and colleagues assessed current American Pediatric Surgical Association (APSA) members with NIH funding. They found that the absolute number of pediatric surgeons with funding had remained relatively steady over the last decade. The accompanying study by Wayne et al. would suggest that the absolute number of pediatric surgeons with active funding has actually increased over the last decade (37 in 2012 compared to 52 at present), but because the APSA membership has grown significantly, that the percentage of NIH funded APSA members is slightly less than a decade ago (5.9 % in 2012 compared to 4.8 % at present) [6].

Pediatric surgeons have managed to secure a significant amount of funding from the NIH over the past few years, with Wayne et al. demonstrating active awards totaling over $44 million for pediatric surgeons [6]. This success can be attributed to several factors that set pediatric surgery research apart from other surgical specialties. Pediatric surgeons often focus on critical areas that have a significant impact on long-term health outcomes. Research in congenital anomalies, pediatric trauma, and early-onset diseases not only addresses immediate surgical needs but also contributes to the broader understanding of child development and long-term health. These areas are of high priority for the NIH, which aims to fund research that promises substantial public health benefits [7].

The field of pediatric surgery is characterized by a high degree of collaboration [8]. Pediatric surgeons frequently partner with researchers in related fields such as pediatrics, oncology, and genetics. These interdisciplinary collaborations enhance the scope and impact of their research, making it more attractive to funding bodies like the NIH. Pediatric surgeons are also at the forefront of adopting and developing innovative surgical techniques and technologies. From minimally invasive procedures to cutting-edge genetic therapies, their research often involves pioneering approaches that push the boundaries of modern medicine. The NIH is particularly interested in funding such groundbreaking work, which can lead to significant advancements in patient care.

## Conclusion

Despite conflicting reports of decreasing NIH funding for surgeon scientists as a whole, pediatric surgeons have demonstrated remarkable resilience and success in securing significant extramural funds from the NIH. Their focus on high-priority research areas, collaborative efforts, and innovative approaches have made their work particularly compelling to funding bodies. Additionally, pediatric surgeons may have lower rates of external pressure related to clinical demands. As a result, pediatric surgeon scientists contribute to the overall advancement of medical science and work to improve outcomes for children.

As work continues to define the current landscape of the surgeon scientist, readers should again be cautioned that different methodologies and inclusion criteria may alter the results from one study to the next. Although there currently appears to be a stable absolute number of pediatric surgeon scientists, increases in the number of pediatric fellowship training positions, academic faculty positions, and of APSA membership as a whole increase the denominator, and should caution the pediatric surgical community that greater percentages of individuals may not actually be pursuing careers in pediatric surgical research. Finding novel strategies to preserve the pediatric surgeon scientist in the current era should therefore be undertaken.

## Credit authorship contribution statement

**Troy A. Markel:** Conceptualization, Writing – original draft, Writing – review & editing.

## Ethical approval statement

This work did not involve human subjects or animals. Therefore, approval from an IRB or IACUC was not required.

## Funding sources

NIDDK R01DK133418, Chan Zuckerberg Initiative 2022-316749, NICHD R01HD105301, IUSM Showalter Scholar Award, Indiana University Bridge Grant, Heartland Grant.

## Declaration of competing interest

I have nothing to declare.
